# Full-length Ebola glycoprotein accumulates in the endoplasmic reticulum

**DOI:** 10.1186/1743-422X-8-11

**Published:** 2011-01-12

**Authors:** Suchita Bhattacharyya, Thomas J Hope

**Affiliations:** 1 Department of Cell and Molecular Biology, Feinberg School of Medicine, Northwestern University, 303 East Chicago Avenue, Chicago, Illinois 60611, USA; 2 Current Address: Nomis Center for Immunobiology and Microbial Pathogenesis, The Salk Institute for Biological Studies, 10010 North Torrey Pines Road, La Jolla, California 92037, USA

## Abstract

The *Filoviridae *family comprises of Ebola and Marburg viruses, which are known to cause lethal hemorrhagic fever. However, there is no effective anti-viral therapy or licensed vaccines currently available for these human pathogens. The envelope glycoprotein (GP) of Ebola virus, which mediates entry into target cells, is cytotoxic and this effect maps to a highly glycosylated mucin-like region in the surface subunit of GP (GP1). However, the mechanism underlying this cytotoxic property of GP is unknown. To gain insight into the basis of this GP-induced cytotoxicity, HEK293T cells were transiently transfected with full-length and mucin-deleted (Δmucin) Ebola GP plasmids and GP localization was examined relative to the nucleus, endoplasmic reticulum (ER), Golgi, early and late endosomes using deconvolution fluorescent microscopy. Full-length Ebola GP was observed to accumulate in the ER. In contrast, GPΔmucin was uniformly expressed throughout the cell and did not localize in the ER. The Ebola major matrix protein VP40 was also co-expressed with GP to investigate its influence on GP localization. GP and VP40 co-expression did not alter GP localization to the ER. Also, when VP40 was co-expressed with the nucleoprotein (NP), it localized to the plasma membrane while NP accumulated in distinct cytoplasmic structures lined with vimentin. These latter structures are consistent with aggresomes and may serve as assembly sites for filoviral nucleocapsids. Collectively, these data suggest that full-length GP, but not GPΔmucin, accumulates in the ER in close proximity to the nuclear membrane, which may underscore its cytotoxic property.

## Findings

Ebola GP is the only viral protein expressed on the virus surface and mediates entry into target cells [1], [2]. However, several studies report that GP expression also causes cell rounding and cytotoxicity, although the underlying mechanism remains unknown. For instance, expression of Ebola GP but not Marburg GP is reported to cause cell detachment without death [3]. Additionally, Ebola GP from Zaire, Sudan and Ivory Coast subtypes are shown to cause cell rounding and detachment ascribed to down-regulation of MHC class I and cell surface adhesion proteins [4], [5]. Interestingly, Ebola GP from the Reston subtype, believed to be non-pathogenic to humans, had a less severe cell rounding effect [4]. GP is also believed to be a key determinant of viral pathogenesis and virus-like particles (VLPs) containing GP are shown to activate human endothelial cells and macrophages [6], [7]. Importantly, the mucin-like region in GP1 is specifically shown to induce cytotoxicity when GP is expressed at similar levels to that seen during Ebola virus infection. Additionally, the other virus proteins tested were not cytotoxic [8]. Collectively, these reports indicate that Ebola GP imparts cell rounding and cytotoxicity in addition to facilitating viral entry.

However, separate work reports that Ebola Zaire GP is not cytotoxic when expressed in isolation at similar levels to that seen during early virus infection [9]. Another study shows that GP is not detected in cells infected with Ebola Zaire virus [10]. This failure to detect GP during infection may arise as GP is released from the infected cells either as soluble glycoprotein (sGP) or a soluble form of GP1 [11]. As full-length GP but not sGP is shown to cause cytotoxicity [12], this suggests that the release of sGP during Ebola virus infection could be a mechanism used by the virus to prevent cytotoxicity and replicate and spread throughout the body. Moreover, this release of sGP may also explain why Ebola Zaire GP expressed at levels similar to early infection is not cytotoxic [9].

Previous studies suggest that Ebola GP is incorporated into VLPs along with the viral VP40 and NP proteins when co-expressed in cells [13], [14], [15]. VP40 is the major matrix protein of Ebola and can drive the formation of filamentous VLPs that resemble wildtype Ebola virus morphology [13]. VP40 plays an important role in viral replication, assembly and budding [16]. VP40 interacts with cellular factors such as the Nedd4 ubiquitin ligase, Tsg101 that comprises part of the ESCRT-I complex, and Sec24C that is a component of the COPII complex [17], [18], [19]. VP40 also has RNA binding and oligomerization properties [20]. The Ebola NP is the principal component of the ribonucleocapsid, which encloses the RNA [21] and is phosphorylated [22].

As the majority of studies suggest a critical role of Ebola GP in causing cytotoxicity [3], [4], [8], [5], [23], [24], and GP interacts with VP40 and NP to form viral particles [13], [14], [15], we therefore investigated the cellular localization of GP, VP40 and NP when transiently expressed in HEK293T cells. Since Ebola GP induces cell rounding and detachment 24 hours after transfection [8], the cellular localization of Ebola GP was examined here 24 hours after transient transfection to try gain insight into the mechanism of GP cytotoxicity.

To this end, HEK293T cells were transiently transfected for 24 hours using the calcium phosphate transfection method [25] with various plasmids. To compare wildtype GP and GPΔmucin localization, 10 μg full-length Ebola Zaire GP (pCB6-EbGP) [2] or GPΔmucin (pCDNA6-EbGPΔmucin-mutΔ1234) [4] were transfected. Their localization relative to cellular ER and Golgi were examined by transfecting 8 μg GP or GPΔmucin with 2 μg pDsRed2-ER or pEYFP-Golgi (Clontech). GP, VP40 and NP localization when expressed in varying combinations were examined by transfecting 5 μg eGFP-VP40 [26] and 5 μg GP or NP plasmids, or 10 μg NP plasmid (pWRG7077-NP) [27] alone. Cells were fixed 24 hours post-transfection and stained. Stains included the Hoechst DNA stain and antibodies targeting nuclear pore complex (NPC) proteins, early or late endosomes. GP was stained with a neutralizing human monoclonal antibody (KZ52) labeled with a Zenon labeling kit (Molecular Probes). Cells were imaged using a DeltaVision microscope with subsequent deconvolution as previously described [28].

**Figure 1:**
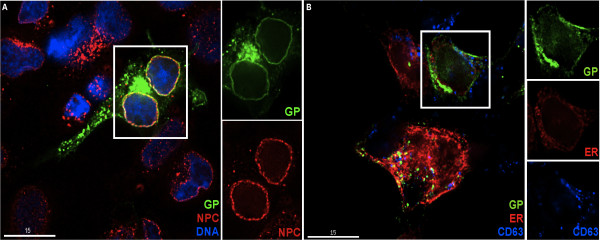
**Full-length Ebola GP localizes in the ER in close proximity to the nuclear membrane**. A. HEK293T cells were transiently transfected with 10 μg GP (pCB6-EbGP) plasmid for 24 hours. Cells were fixed and stained for DNA (*blue*) using Hoechst, nuclear pore complex (NPC, *red*) proteins using mouse monoclonal antibody 414 (Covance Research Products), and GP (*green*) using the neutralizing human monoclonal antibody (KZ52) labeled with a Zenon labeling kit (Molecular Probes). Scale bar represents 15 μm. Side panels show individual fluorescent channels from the boxed region in the image. B. HEK293T cells were transiently transfected for 24 hours with 8 μg GP and 2 μg pDsRed2-ER vector (Clontech) that labels the endoplasmic reticulum (ER, *red*). Cells were fixed and stained for late endosomes using a mouse monoclonal antibody targeting CD63 (BD Biosciences, *blue*), in accordance with a previous publication [43]. GP was stained as above (*green*). Scale bar represents 15 μm. Side panels show individual fluorescent channels from the boxed region in the image.

Full-length Ebola GP localized in close proximity to the nuclear membrane (Figure 1A). However, NPC staining showed little overlap with GP suggesting GP was not localized on the nuclear membrane. Thus, we hypothesized that GP may localize within the ER. Co-expression of Ebola GP with DsRed2-ER showed that GP had localized within the ER (Figure 1B). Notably, GP was not found within late endosomes (Figure 1B), Golgi or early endosomes (data not shown).

**Figure 2:**
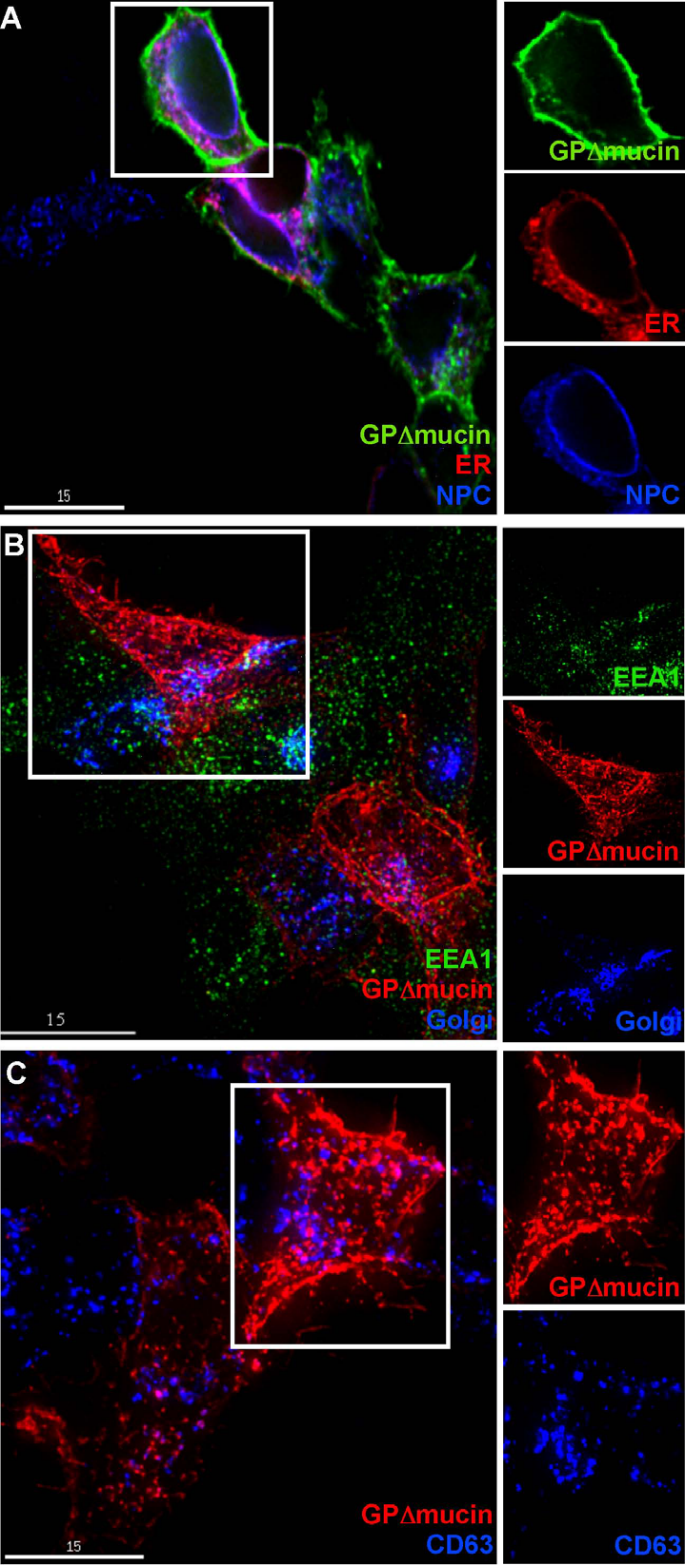
**Ebola GP lacking the mucin-like region does not accumulate in the ER**. A. HEK293T cells were transiently transfected for 24 hours with 8 μg GPΔmucin (pCDNA6-EbGPΔmucin-mutΔ1234) and 2 μg pDsRed2-ER vector (Clontech) that labels the endoplasmic reticulum (ER, *red*). Cells were fixed and stained for NPC proteins (*blue*) using mouse monoclonal antibody 414 (Covance Research Products) and GPΔmucin (*green*) using the KZ52 neutralizing human monoclonal antibody labeled with a Zenon labeling kit (Molecular Probes). Scale bar represents 15 μm. Side panels show individual fluorescent channels from the boxed region in the image. B. HEK293T cells were transiently transfected for 24 hours with 8 μg GPΔmucin and 2 μg pEYFP-Golgi vector (Clontech) that labels the trans-medial region of the Golgi apparatus (*blue*). Cells were fixed and stained for early endosomes using a mouse monoclonal antibody against EEA1 (BD Biosciences, *green*) and for GPΔmucin as above (*red*). Scale bar represents 15 μm. Side panels show individual fluorescent channels from the boxed region in the image. C. HEK293T cells were transiently transfected with 10 μg GPΔmucin for 24 hours. Cells were fixed and stained for late endosomes using a mouse monoclonal antibody targeting CD63 (BD Biosciences, *blue*) and for GPΔmucin as above (*red*). Scale bar represents 15 μm. Side panels show individual fluorescent channels from the boxed region in the image.

Since full-length GP localized within the ER in close proximity to the nuclear membrane, we then examined whether GP lacking the mucin-like region and cytotoxic activity [4], [8] also localized in the ER. Strikingly, while GPΔmucin was expressed at comparable levels to full-length GP, it did not accumulate in the ER (Figure 2A). Instead, GPΔmucin was uniformly expressed throughout the cell and did not localize within Golgi, early endosomes (Figure 2B), or late endosomes (Figure 2C) either. This dispersed localization suggests that it is diffusely localized in the plasma membrane.

**Figure 3:**
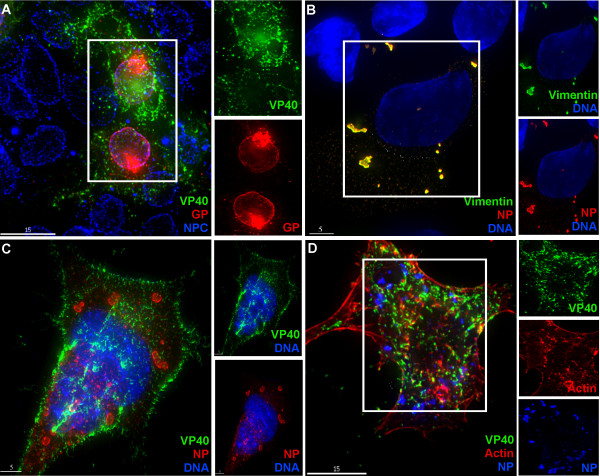
**Co-expression of Ebola GP, VP40 and NP in HEK293T cells does not alter localization of individual proteins**. A. HEK293T cells were co-transfected with 5 μg Ebola GP and 5 μg eGFP-VP40 plasmids for 24 hours. Cells were fixed and stained for NPC proteins using mouse monoclonal antibody 414 (Covance Research Products, *blue*) and GP (*red*) using KZ52 neutralizing human monoclonal antibody labeled with a Zenon labeling kit (Molecular Probes). Green represents VP40. Scale bar represents 15 μm. Upper and lower side panels show VP40 and GP fluorescence respectively, from the boxed region in the image. B. HEK293T cells were transfected with 10 μg Ebola NP plasmid for 24 hours. Cells were fixed and stained for DNA using Hoechst (*blue*), NP using a mouse monoclonal antibody targeting NP (*red*) and vimentin using a mouse monoclonal antibody targeting vimentin (*green*). Scale bar represents 5 μm. Upper and lower side panels show the vimentin and DNA fluorescence and the NP and DNA fluorescence respectively, from the boxed region in the image. C. HEK293T cells were co-transfected with 5 μg Ebola NP and 5 μg eGFP-VP40 plasmids for 24 hours. Cells were fixed and stained for NP using a mouse monoclonal antibody targeting NP (*red*) and DNA using Hoechst (*blue*). Green represents VP40. Scale bar represents 5 μm. Upper and lower side panels show the VP40 and DNA fluorescence and the NP and DNA fluorescence respectively. D. HEK293T cells were co-transfected with 5 μg Ebola NP and 5 μg eGFP-VP40 plasmids for 24 hours. Cells were fixed and stained for NP using a mouse monoclonal antibody targeting NP (*blue*) and filamentous actin using Texas Red Phalloidin (Invitrogen, *red*). Green represents VP40. Scale bar represents 15 μm. Side panels show the individual fluorescent channels from the boxed region in the image.

Co-expression of GP and VP40 did not alter the localization of either protein. GP remained in close proximity to the nuclear membrane consistent with an ER localization while VP40 localized near the plasma membrane (Figure 3A), which agrees with previous reports [29], [30], [31]. However, separate studies show that Ebola GP localizes in the plasma membrane of either tissues from experimentally infected non-human primates [10] or HeLa cells 48 hours post-transfection [24]. Additionally, GP is reported to localize within VP40 filamentous structures following GP and VP40 co-expression, suggesting GP interacts with VP40 during morphogenesis [13]. While we observed little overlay between GP and VP40 here, GP must associate with VP40 filaments to produce infectious virions perhaps during a later phase of the viral replication cycle. Therefore at this early 24 hour time-point, it is likely the GP amount associated with the VP40 filamentous structures is limited compared to the total cellular amount of GP, making it difficult to visualize by fluorescence microscopy here.

Ebola NP expressed in isolation accumulated in large cytoplasmic aggregates (Figure 3B). Staining for the intermediate filament protein, vimentin revealed these NP aggregates were lined with vimentin. Aggresomes are perinuclear structures lined with vimentin, which recruit molecular chaperones and proteosomes. They are believed to regulate protein folding and degradation of misfolded proteins [32]. Therefore, the NP association with vimentin here suggests the NP was present in aggresome-like structures. Previous studies using cells infected with Marburg virus report Marburg NP accumulates in structures resembling inclusion bodies in close proximity to the ER [33], [34]. So it is possible that the Ebola NP in these aggresome-like structures could perhaps serve as sites for assembly of filoviral nucleocapsid analogous to African swine fever virus [35] and herpes simplex virus type 2 [36].

VP40 and NP did not colocalize upon co-expression (Figure 3C). VP40 was seen as filamentous structures near the plasma membrane, while NP was localized within distinct cytoplasmic aggresome-like bodies. Actin staining also showed little overlay with either VP40 or NP (Figure 3D), correlating with the report that actin incorporates into VLPs containing both GP and VP40, but not VP40 alone [37]. While we did not detect NP in the VP40 filamentous structures here, previous studies suggest that NP interacts directly with VP40, and is present in VP40-containing VLPs when VP40 and NP are co-expressed [14], [15]. Thus, perhaps NP is recruited to VP40-containing VLPs at a later stage during filament formation than the 24 hours experiment here, or another viral protein is required for VP40 and NP interaction.

As filoviral replication takes place in the cytoplasm [38], it is intriguing that full-length GP but not GPΔmucin, accumulates in the ER in close proximity to the nuclear membrane after 24 hours transient expression in cells. This suggests that full-length GP localization in the ER could play a role in its cytotoxic and cell rounding properties because the mucin-like region of GP is reported to cause cytotoxicity [4], [8].

How accumulation of GP in the ER might induce cytotoxic effects remains to be defined. The GP mucin-like region activates the NFκB signaling pathway [39] and also downregulates activation of the MAPK effector ERK2, which is linked to GP induced cytotoxicity [40]. Recently, the Classical Swine Fever virus NS2 protein is reported to localize to the ER and cause ER stress and NFκB activation [41]. Similarly, the SARS coronavirus 3a protein causes ER stress and activates the PERK pathway leading to unfolded protein response (UPR) [42]. Therefore, Ebola GP accumulation in the ER may interfere with protein synthesis, folding and transport thereby activating UPR to cause ER stress. Understanding the exact mechanism of GP accumulation in the ER and its correlation to cytotoxicity may be useful in designing inhibitors to block this cytotoxic effect during Ebola virus infection of patients and/or potentially reduce the severe pathogenic effects these patients experience.

## List of abbreviations

EbGP: Ebola virus glycoprotein; ER: endoplasmic reticulum; ERK2: mitogen responsive extracellular regulated kinase 2; GP1: surface subunit of GP; GPΔmucin: GP with the mucin-like region of GP1 deleted; MAPK: mitogen activated protein kinase; NP: nucleoprotein; NPC: nuclear pore complex; PERK: PRKR-like ER kinase; sGP: soluble glycoprotein; UPR: unfolded protein response; VLP: virus-like particle.

## Competing interests

The authors declare that they have no competing interests.

## Authors' contributions

SB and TJH conceived and designed the study, SB performed the experiments, and SB and TJH interpreted the results and wrote the manuscript. All authors read and approved the final manuscript.

## References

[B1] Takada A Robison C Goto H Sanchez A Murti KG Whitt MA Kawaoka Y A system for functional analysis of Ebola virus glycoprotein Proc Natl Acad Sci USA 1997 94 14764 14769 10.1073/pnas.94.26.14764 9405687 PMC25111

[B2] Wool-Lewis RJ Bates P Characterization of Ebola virus entry by using pseudotyped viruses: identification of receptor-deficient cell lines J Virol 1998 72 3155 3160 9525641 10.1128/jvi.72.4.3155-3160.1998 PMC109772

[B3] Chan SY Ma MC Goldsmith MA Differential induction of cellular detachment by envelope glycoproteins of Marburg and Ebola (Zaire) viruses J Gen Virol 2000 81 2155 2159 10950971 10.1099/0022-1317-81-9-2155

[B4] Simmons G Wool-Lewis RJ Baribaud F Netter RC Bates P Ebola virus glycoproteins induce global surface protein down-modulation and loss of cell adherence J Virol 2002 76 2518 2528 10.1128/jvi.76.5.2518-2528.2002 11836430 PMC153797

[B5] Takada A Watanabe S Ito H Okazaki K Kida H Kawaoka Y Downregulation of beta1 integrins by Ebola virus glycoprotein: implication for virus entry Virology 2000 278 20 26 10.1006/viro.2000.0601 11112476

[B6] Wahl-Jensen VM Afanasieva TA Seebach J Stroher U Feldmann H Schnittler HJ Effects of Ebola virus glycoproteins on endothelial cell activation and barrier function J Virol 2005 79 10442 10450 10.1128/JVI.79.16.10442-10450.2005 16051836 PMC1182673

[B7] Wahl-Jensen V Kurz SK Hazelton PR Schnittler HJ Stroher U Burton DR Feldmann H Role of Ebola virus secreted glycoproteins and virus-like particles in activation of human macrophages J Virol 2005 79 2413 2419 10.1128/JVI.79.4.2413-2419.2005 15681442 PMC546544

[B8] Yang ZY Duckers HJ Sullivan NJ Sanchez A Nabel EG Nabel GJ Identification of the Ebola virus glycoprotein as the main viral determinant of vascular cell cytotoxicity and injury Nat Med 2000 6 886 889 10.1038/78645 10932225

[B9] Alazard-Dany N Volchkova V Reynard O Carbonnelle C Dolnik O Ottmann M Khromykh A Volchkov VE Ebola virus glycoprotein GP is not cytotoxic when expressed constitutively at a moderate level J Gen Virol 2006 87 1247 1257 10.1099/vir.0.81361-0 16603527

[B10] Steele K Crise B Kuehne A Kell W Ebola virus glycoprotein demonstrates differential cellular localization in infected cell types of nonhuman primates and guinea pigs Arch Pathol Lab Med 2001 125 625 630 11300932 10.5858/2001-125-0625-EVGDDC

[B11] Volchkov VE Volchkova VA Slenczka W Klenk HD Feldmann H Release of viral glycoproteins during Ebola virus infection Virology 1998 245 110 119 10.1006/viro.1998.9143 9614872

[B12] Volchkov VE Volchkova VA Muhlberger E Kolesnikova LV Weik M Dolnik O Klenk HD Recovery of infectious Ebola virus from complementary DNA: RNA editing of the GP gene and viral cytotoxicity Science 2001 291 1965 1969 10.1126/science.1057269 11239157

[B13] Noda T Sagara H Suzuki E Takada A Kida H Kawaoka Y Ebola virus VP40 drives the formation of virus-like filamentous particles along with GP J Virol 2002 76 4855 4865 10.1128/JVI.76.10.4855-4865.2002 11967302 PMC136157

[B14] Licata JM Johnson RF Han Z Harty RN Contribution of ebola virus glycoprotein, nucleoprotein, and VP24 to budding of VP40 virus-like particles J Virol 2004 78 7344 7351 10.1128/JVI.78.14.7344-7351.2004 15220407 PMC434112

[B15] Johnson RF Bell P Harty RN Effect of Ebola virus proteins GP, NP and VP35 on VP40 VLP morphology Virol J 2006 3 31 10.1186/1743-422X-3-31 16719918 PMC1502131

[B16] Feldmann H Klenk HD Sanchez A Molecular biology and evolution of filoviruses Arch Virol Suppl 1993 7 81 100 8219816 10.1007/978-3-7091-9300-6_8

[B17] Yasuda J Nakao M Kawaoka Y Shida H Nedd4 regulates egress of Ebola virus-like particles from host cells J Virol 2003 77 9987 9992 10.1128/JVI.77.18.9987-9992.2003 12941909 PMC224586

[B18] Martin-Serrano J Zang T Bieniasz PD HIV-1 and Ebola virus encode small peptide motifs that recruit Tsg101 to sites of particle assembly to facilitate egress Nat Med 2001 7 1313 1319 10.1038/nm1201-1313 11726971

[B19] Yamayoshi S Noda T Ebihara H Goto H Morikawa Y Lukashevich IS Neumann G Feldmann H Kawaoka Y Ebola virus matrix protein VP40 uses the COPII transport system for its intracellular transport Cell Host Microbe 2008 3 168 177 10.1016/j.chom.2008.02.001 18329616 PMC2330329

[B20] Hoenen T Volchkov V Kolesnikova L Mittler E Timmins J Ottmann M Reynard O Becker S Weissenhorn W VP40 octamers are essential for Ebola virus replication J Virol 2005 79 1898 1905 10.1128/JVI.79.3.1898-1905.2005 15650213 PMC544139

[B21] Sanchez A Kiley MP Holloway BP McCormick JB Auperin DD The nucleoprotein gene of Ebola virus: cloning, sequencing, and in vitro expression Virology 1989 170 81 91 10.1016/0042-6822(89)90354-1 2718390

[B22] Elliott LH Kiley MP McCormick JB Descriptive analysis of Ebola virus proteins Virology 1985 147 169 176 10.1016/0042-6822(85)90236-3 4060597

[B23] Sullivan NJ Peterson M Yang ZY Kong WP Duckers H Nabel E Nabel GJ Ebola virus glycoprotein toxicity is mediated by a dynamin-dependent protein-trafficking pathway J Virol 2005 79 547 553 10.1128/JVI.79.1.547-553.2005 15596847 PMC538691

[B24] Francica JR Matukonis MK Bates P Requirements for cell rounding and surface protein down-regulation by Ebola virus glycoprotein Virology 2009 383 237 247 10.1016/j.virol.2008.10.029 19013626 PMC2654768

[B25] Bartz SR Vodicka MA Production of high-titer human immunodeficiency virus type 1 pseudotyped with vesicular stomatitis virus glycoprotein Methods 1997 12 337 342 10.1006/meth.1997.0487 9245614

[B26] Martin-Serrano J Perez-Caballero D Bieniasz PD Context-dependent effects of L domains and ubiquitination on viral budding J Virol 2004 78 5554 5563 10.1128/JVI.78.11.5554-5563.2004 15140952 PMC415830

[B27] Warfield KL Swenson DL Olinger GG Kalina WV Aman MJ Bavari S Ebola virus-like particle-based vaccine protects nonhuman primates against lethal Ebola virus challenge J Infect Dis 2007 196 Suppl 2 S430 437 10.1086/520583 17940980

[B28] Bhattacharyya S Warfield KL Ruthel G Bavari S Aman MJ Hope TJ Ebola virus uses clathrin-mediated endocytosis as an entry pathway Virology 2010 401 18 28 10.1016/j.virol.2010.02.015 20202662 PMC3732189

[B29] Panchal RG Ruthel G Kenny TA Kallstrom GH Lane D Badie SS Li L Bavari S Aman MJ In vivo oligomerization and raft localization of Ebola virus protein VP40 during vesicular budding Proc Natl Acad Sci USA 2003 100 15936 15941 10.1073/pnas.2533915100 14673115 PMC307671

[B30] Licata JM Simpson-Holley M Wright NT Han Z Paragas J Harty RN Overlapping motifs (PTAP and PPEY) within the Ebola virus VP40 protein function independently as late budding domains: involvement of host proteins TSG101 and VPS-4 J Virol 2003 77 1812 1819 10.1128/JVI.77.3.1812-1819.2003 12525615 PMC140960

[B31] McCarthy SE Johnson RF Zhang YA Sunyer JO Harty RN Role for amino acids 212KLR214 of Ebola virus VP40 in assembly and budding J Virol 2007 81 11452 11460 10.1128/JVI.00853-07 17699576 PMC2045517

[B32] Kopito RR Aggresomes, inclusion bodies and protein aggregation Trends Cell Biol 2000 10 524 530 10.1016/S0962-8924(00)01852-3 11121744

[B33] Kolesnikova L Muhlberger E Ryabchikova E Becker S Ultrastructural organization of recombinant Marburg virus nucleoprotein: comparison with Marburg virus inclusions J Virol 2000 74 3899 3904 10.1128/JVI.74.8.3899-3904.2000 10729166 PMC111900

[B34] Becker S Rinne C Hofsass U Klenk HD Muhlberger E Interactions of Marburg virus nucleocapsid proteins Virology 1998 249 406 417 10.1006/viro.1998.9328 9791031

[B35] Heath CM Windsor M Wileman T Aggresomes resemble sites specialized for virus assembly J Cell Biol 2001 153 449 455 10.1083/jcb.153.3.449 11331297 PMC2190574

[B36] Nozawa N Yamauchi Y Ohtsuka K Kawaguchi Y Nishiyama Y Formation of aggresome-like structures in herpes simplex virus type 2-infected cells and a potential role in virus assembly Exp Cell Res 2004 299 486 497 10.1016/j.yexcr.2004.06.010 15350546

[B37] Han Z Harty RN Packaging of actin into Ebola virus VLPs Virol J 2005 2 92 10.1186/1743-422X-2-92 16367999 PMC1334228

[B38] Feldmann H Kiley MP Classification, structure, and replication of filoviruses Curr Top Microbiol Immunol 1999 235 1 21 9893375 10.1007/978-3-642-59949-1_1

[B39] Martinez O Valmas C Basler CF Ebola virus-like particle-induced activation of NF-kappaB and Erk signaling in human dendritic cells requires the glycoprotein mucin domain Virology 2007 364 342 354 10.1016/j.virol.2007.03.020 17434557 PMC2034500

[B40] Zampieri CA Fortin JF Nolan GP Nabel GJ The ERK mitogen-activated protein kinase pathway contributes to Ebola virus glycoprotein-induced cytotoxicity J Virol 2007 81 1230 1240 10.1128/JVI.01586-06 17108034 PMC1797502

[B41] Tang QH Zhang YM Fan L Tong G He L Dai C Classic swine fever virus NS2 protein leads to the induction of cell cycle arrest at S-phase and endoplasmic reticulum stress Virol J 2010 7 4 10.1186/1743-422X-7-4 20064240 PMC2819037

[B42] Minakshi R Padhan K Rani M Khan N Ahmad F Jameel S The SARS Coronavirus 3a protein causes endoplasmic reticulum stress and induces ligand-independent downregulation of the type 1 interferon receptor PLoS One 2009 4 e8342 10.1371/journal.pone.0008342 20020050 PMC2791231

[B43] Kobayashi T Beuchat MH Lindsay M Frias S Palmiter RD Sakuraba H Parton RG Gruenberg J Late endosomal membranes rich in lysobisphosphatidic acid regulate cholesterol transport Nat Cell Biol 1999 1 113 118 10.1038/15666 10559883

